# Retraction Note: NT-4 attenuates neuroinflammation via TrkB/PI3K/FoxO1 pathway after germinal matrix hemorrhage in neonatal rats

**DOI:** 10.1186/s12974-025-03681-3

**Published:** 2026-01-22

**Authors:** Tianyi Wang, Junyi Zhang, Peng Li, Yan Ding, Jiping Tang, Gang Chen, John H. Zhang

**Affiliations:** 1 Department of Physiology and Pharmacology, School of Medicine, Loma Linda University, 11041 Campus Street, Loma Linda, CA 92350 USA; 2 Department of Neurosurgery, The First Affiliated Hospital of Soochow University, Suzhou, 215000 China

**Retraction Note: J Neuroinflammation 17, 158 (2020)**

**https://doi.org/10.1186/s12974-020-01835-z**

The Editors-in-Chief have retracted this article. After publication, concerns were raised regarding highly similar images within the same article. Specifically:

Fig. 6A, p-AKT (lane 5-6-7) and Fig.7A, p-AKT (lane 1-2-3) appear to be highly similar

Fig. 3A, Actin and Fig.6, Actin (lane 1-2-3) appear to be highly similar

The Authors confirmed the similarities but upon request, were unable to provide original raw data. Therefore, the Editors-in-Chief no longer have confidence in the presented data.

Zhang J.H. disagrees with this retraction. Wang T., Zhang J., Ding Y., and J., Chen G. did not respond to correspondence from the Publisher about this retraction and retraction notice.

